# Chinese Birch Pollen Allergy and Immunotherapy in Mice

**DOI:** 10.1007/s10753-019-00957-8

**Published:** 2019-02-04

**Authors:** Zhijuan Xie, Jia Yin

**Affiliations:** Department of Allergy, Peking Union Medical College Hospital, Chinese Academy of Medical Sciences and Peking Union Medical College, No. 1 Shuai Fu Yuan, Wang Fu Jing Street, Beijing, 100730 China

**Keywords:** Chinese birch pollen allergy, birch pollen allergen, mice model, asthma, 70-kDa allergen

## Abstract

Birch pollen allergy is a common cause of spring pollinosis in China. However, there is little research on birch pollen allergen in China and only the major allergen (Bet v 1) has been fully characterized. Chinese birch pollen-induced airway inflammation models in BALB/c mice were developed and administered subcutaneous immunotherapy (SCIT). BALB/c mice were sensitized subcutaneously on days 1, 8, and 15 with 25 μg/μL birch pollen extract. On days 24–26, the mice were challenged with 0.1% birch pollen aerosol. To investigate the efficacy of SCIT, mice were subcutaneously injected 0.3 mg birch pollen extract (BPE) with or without being adsorbed to alum. Airway hyper-responsiveness (AHR) to methacholine and immunological parameters was detected. Western blot analysis was applied with mice serum and mass spectrometry was used to identify the IgE-binding bands in birch pollen. Compared with PBS group, birch pollen sensitization and challenge BALB/c mice developed AHR, and IL4, IL5, IL6, IL10, and IL17 were significantly higher. Mice sensitized by birch pollen showed increased plasma levels of anti-BPE IgE, IgG1, and IgG2a. Histologic analyses showed that mice had peribranchial infiltration of inflammatory cells and mucosal hyperplasia. After SCIT, allergic symptoms effectively alleviated and kept for a long time. Interestingly, mice serum pool showed strong reactions to 70-kDa proteins. Mass spectrometry data suggests that the 70-kDa protein belongs to the HSP 70 family. SCIT inhibited the inflammatory response in the long term and a 70-kDa protein potentially belonging to the HSP 70 family plays a significant role in Chinese birch pollen-induced mice model.

## INTRODUCTION

Birch pollen is one of the most important spring pollens in north of China. In Europe, 70% of birch pollen allergy patients have accompanying varying degrees of food allergy [1]. Their symptoms are mainly shown after ingesting birch pollen-related foods [2], at least in part because of the cross-reaction between Bet v 1 homologous protein in food and the major allergen Bet v 1 in birch pollen, including apple (Mal d 1), cherry (Pru av. 1), pear (Pyr c 1), hazelnut (Cor a 1), celery (Api g 1), carrot (Dau c 1), soybean (Gly m 4), peanut (Ara h 8), jackfruit, and kiwi (Act d 8) [3].

Subcutaneous immunotherapy (SCIT) is the only way to effectively control allergy symptoms and it can change the natural courses from allergic rhinitis to allergic asthma [4–7]. They demonstrated that the mechanism of SCIT is largely related to the reduction of Th2-related cytokines (IL4 and IL5) and Th17-related cytokines (IL17). It is also thought that SCIT induces the production of protective antibodies IgG4 (IgG2a in mice) to inhibit mast cell activation [8, 9]. Regulatory T cells (Treg) suppress the allergic inflammation by producing IL10 and TGF-β in SCIT. IL-10 suppresses T cell responses, promotes IgG4 production, and suppresses IgE production [10]. TGF-β is required for Treg differentiation. Moreover, IL-6 may regulate the balance between Th17 cells and Treg cells [11]. Adjuvants, especially the alum, play a central role in co-stimulating immune cells in the SCIT, which also lead to local or systemic adverse effects.

Seven allergens in birch pollen have been included in the official allergen list of the World Health Organization and International Union of Immunological Societies (WHO/IUIS) allergen nomenclature subcommittee (www.allergen.org), named sequentially as Bet v 1 through Bet v 7. Research is often biased toward a single major allergen (Bet v 1) [12]. Panallergens are ubiquitous with broad cross-sensitization and underlie certain pollen-food or plant-food syndromes [13]. However, there is little research conducted on birch pollen allergen in China.

In this study, it is the first time to establish an allergy murine model with Chinese birch pollen and observe the long efficacy of SCIT in mice. We also hypothesized that SCIT without adjuvant (alum) remains efficient in mice.

## MATERIALS AND METHODS

### Mice

Female BALB/c mice, at 5–6 weeks of age, were purchased from Beijing Vital River Laboratory Animal Technology Co, Beijing, China. All of the mice were given a 7-day period acclimate. The animals were kept under Specific Pathogen Free (SPF) laboratory conditions in the Center for Animal Experiments of Peking Union Medical College Hospital. The institutional animal ethics committee approved all experiments.

### Allergens

Standard birch pollen extracts (BPE) were provided by the department of allergy, Peking Union Medical College Hospital, Beijing, China. These can be used for patients in clinics. The protein concentration was determined using the Pierce BCA Protein Assay Kit (Thermo Fisher Scientific, Waltham, Mass). The major protein Bet v1 of birch pollen extract was determined by a Bet v 1 ELISA kit (4 e10 b10/2) (Indoor Biotechnologies Inc., Cardiff, UK).

### Study Design

#### Allergen Sensitization

**Fig. 1:**
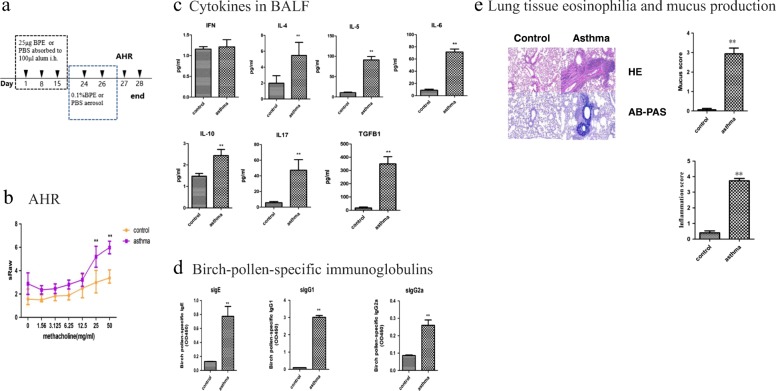
Allergen sensitization. **A** Timeline for female mice with BPE. BALB/c mice were sensitized subcutaneously with 25 μg/μl BPE or PBS on days 1, 8, and 15. On days 24–26, the mice were challenged by nebulization with 0.1% birch pollen aerosol or PBS aerosol. After 1 day, AHR to methacholine was assessed. After 24 h, mice were euthanized. **B** Airway hyper-reactivity to methacholine is shown on day 27. **C** Cytokines in BALF are shown. **D** Birch pollen-specific immunoglobulins (sIgE, sIgG1, sIgG2a) are measured by using ELISA. **E** Lung tissue eosinophilia and mucus production are shown. Lung specimens were stained with hematoxylin and eosin (H&E) and AB-PAS staining. Data are shown as means ± SD from 8 to 10 mice per group, ** represents the asthma group *vs.* the control group, *P* < 0.01; * represents the asthma group *vs.* the control group, *P* < 0.05.

BALB/c mice were sensitized subcutaneously on days 1, 8, and 15 with 25 μg/μL birch pollen extracts, which contained 5 μg Bet v 1, absorbed to 100 μL alum (Imject Alum, Thermo, USA) by Eppendorf plus. On days 24–26, the mice were challenged with 0.1% birch pollen extract aerosol. After 1 day, airway hyper-responsiveness (AHR) to methacholine was assessed. The control group were sensitized subcutaneously with Phosphate Buffered Saline (PBS) and challenged with PBS. (Fig. 1A).

### SCIT Treatment

**Fig. 2:**
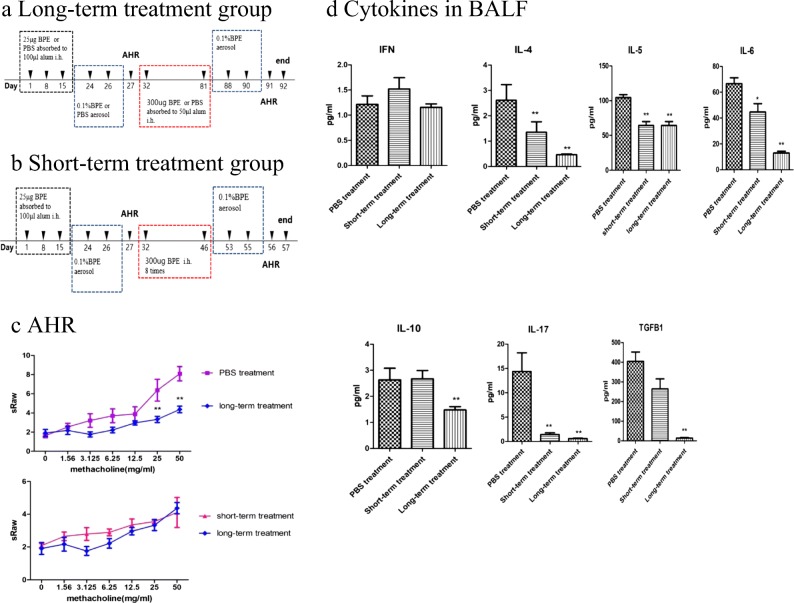

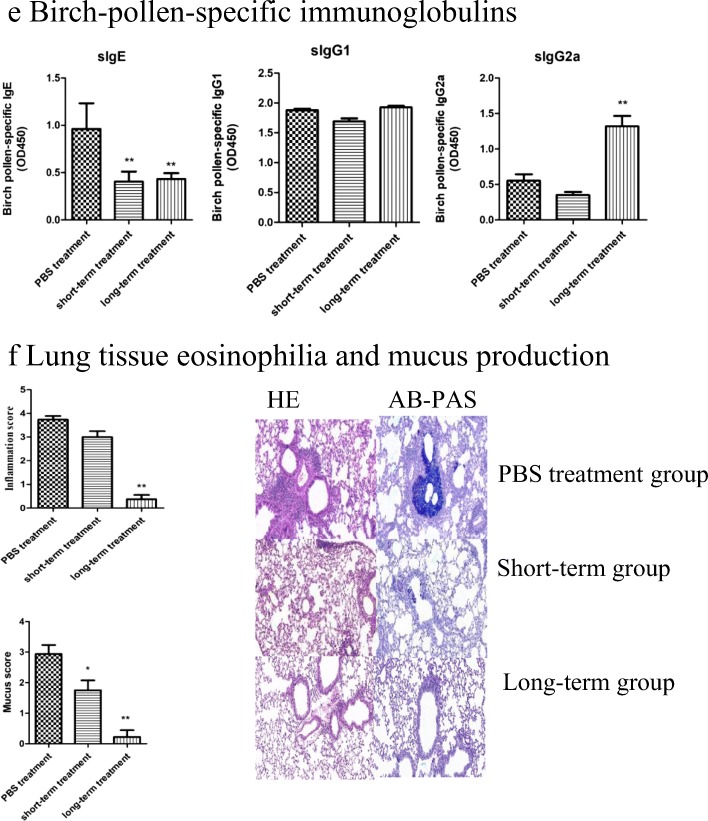
SCIT provides therapeutic benefit. **A** Timeline for the long-term treatment group. BPE was injected subcutaneously on days 1, 8, and 15. On days 24–26, the mice were challenged with 0.1% birch pollen aerosol. After 1 day, AHR to methacholine was assessed. Mice were subcutaneously injected with 0.3 mg BPE adsorbed to 0.1 mg alum on days 32, 39, 46, 53, 60, 67, 74, and 81. On days 88–90, the mice were re-challenged for three consecutive days with 0.1% birch pollen extract aerosol, 30 min per day, as before. After 1 day, AHR to methacholine was assessed. After 24 h, the mice were euthanized for evaluation using the immunological parameters. The PBS treatment group: BPE was injected subcutaneously on days 1, 8, and 15. On days 24–26, the mice were challenged with 0.1% birch pollen aerosol. After 1 day, AHR to methacholine was assessed. Mice were subcutaneously injected with PBS adsorbed to 0.1 mg alum on days 32, 39, 46, 53, 60, 67, 74, and 81. On days 88–90, the mice were re-challenged for three consecutive days with 0.1% birch pollen extract aerosol, 30 min per day. After 1 day, AHR to methacholine was assessed. After 24 h, the mice were euthanized for evaluation using the immunological parameters. **B** Timeline for the short-term treatment group. After allergen sensitization, mice were subcutaneously injected with 0.3 mg birch pollen extract (BPE) without being adsorbed to alum on days 32, 34, 36, 38, 40, 42, 44, and 46. Then, the mice were challenged as described above. AHR to methacholine was evaluated on day 56. The next day, the mice were euthanized and evaluated. **C** Airway hyper-reactivity to methacholine is shown. The PBS treatment group and the long-term treatment group; the short-term treatment group and the long-term treatment group. **D** Cytokines in BALF are shown. **E** Birch pollen specific immunoglobulins (sIgE, sIgG1, sIgG2a) are measured by using ELISA. **F** Lung tissue eosinophilia and mucus production are shown. Lung specimens were stained with hematoxylin and eosin (H&E) and AB-PAS staining. Data are shown as means ± SD, ** represents the short-term treatment group and the long-term treatment group *vs.* the PBS treatment group, *P* < 0.01; * represents the short-term treatment group and the long-term treatment group *vs.* the PBS treatment group, *P* < 0.05.

The long-term treatment group: mice were subcutaneously injected with 0.3 mg BPE adsorbed to 0.1 mg alum on days 32, 39, 46, 53, 60, 67, 74, and 81. On days 88–90, the mice were re-challenged for three consecutive days with 0.1% BPE aerosol, 30 min per day, as before. After 1 day, AHR to methacholine was assessed. After 24 h, the mice were euthanized for evaluation using the immunological parameters. The protocol was described by past studies [14] (Fig. 2A).

The short-term treatment group: mice were subcutaneously injected with 0.3 mg birch pollen extract (BPE) without being adsorbed to alum on days 32, 34, 36, 38, 40, 42, 44, and 46. Then, the mice were challenged with 0.1% BPE aerosol. AHR to methacholine was evaluated on day 56. The next day, the mice were euthanized and evaluated (Fig. 2B).

#### Observation

**Fig. 3:**
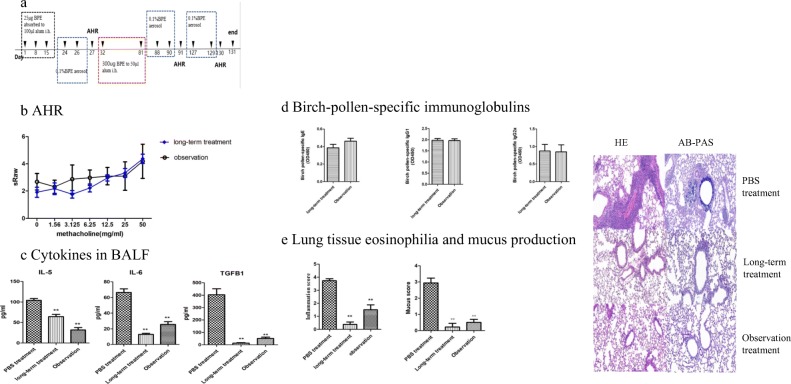
Long-term efficacy of SCIT. **A** Timeline for observation group. After the long-term treatment, mice were kept without any treatment on days 92–126. Then, the mice were challenged again for another three consecutive days. After 1 day, AHR to methacholine was assessed. After 24 h, the mice were euthanized and serum was collected. **B** Airway hyper-reactivity to methacholine is shown; the observation group *vs.* the long-term treatment group. **C** Cytokines in BALF are shown. **D** Birch pollen specific immunoglobulins (sIgE, sIgG1, sIgG2a) are measured by using ELISA. **E** Lung tissue eosinophilia and mucus production are shown. Lung specimens were stained with hematoxylin and eosin (H&E) and AB-PAS staining. Data are shown as means ± SD, ** represents the observation group *vs.* the long-term treatment group, *P* < 0.01; * represents the observation group *vs.* the long-term treatment group, *P* < 0.05.

In order to evaluate the long-term efficacy of SCIT, mice were kept without any treatment on days 92–126 after receiving the long-term treatment. Then, the mice were challenged again for another three consecutive days. After 1 day, AHR to methacholine was assessed. After 24 h, the mice were euthanized and serum was collected (Fig. 3A). The immunological parameters were compared with the long-term treatment.

In the treatment phase, the PBS treatment group were sensitized, challenged, and non-treated mice. It means the mice were sensitized subcutaneously with BPE, challenged with BPE, and received PBS treatment.

### Airway Hyper-responsiveness (AHR) Measurement

Airway responsiveness was measured in mice 24 h after the final birch pollen challenge with a Buxco FinePointe System for noninvasive airway measurement (Buxco, Wilmington, USA) according to the manufacturer’s instructions. Mice were given 5 min to acclimate and then exposed to aerosolized PBS to set a baseline value, followed by increasing concentrations of 100 μl aerosolized methacholine (3.125, 6.25, 12.5, 25, and 50 mg/mL in PBS for 1 min; Sigma-Aldrich, German). The specific airway resistance (sRAW) were measured after each methacholine aerosol application by the noninvasive airway device.

### Cytokine Evaluation

In brief, the bronchial tube was lavaged three times with 0.8 mL of PBS *via* a tracheal cannula and then the bronchoalveolar lavage fluid (BALF) was recovered. The BALF were centrifuged at 4000 RPM for 10 min at 4 °C. The supernatant was stored at − 80 °C until the measurement of cytokines. IL-4, IL-10, IL-17A, and IFN-γ in the supernatant of BALF were determined by the MILLIPLEX MAP Mouse Cytokine/Chemokine Magnetic Panel (Millipore, Billerica, USA) according to manufacturer’s instructions. The results were analyzed with the Bio-Plex System (Bio-Rad Laboratories, Hercules, USA). Values were reported in pg/ml. IL-5, IL-6, and TGF-β levels in the BALF were measured with ELISA kits (eBioscience, San Diego, USA), according to the protocol recommended by the manufacturer.

### Birch Pollen Specific Serum IgE, IgG1, and IgG2a

Serum levels of birch pollen IgE, IgG1, and IgG2a were measured by Enzyme-Linked Immunosorbent Assay (ELISA). Briefly, 96-well plates (Thermo Fisher Scientific, Waltham, USA) were coated with 100 μl/well birch pollen extract (0.05 mg/mL) diluted with PBS overnight at 4 °C. Then, washed the plate for three times, added 200 μl blocking buffer (5% skim milk in PBS) to incubate for 2 h at room temperature. After another three washes, serum samples were diluted 1/200 for IgG1, 1/200 for IgG2a, and 1/10 for IgE with PBS and 100 μl/well was added to the plate overnight at 4 °C. The serum samples were washed again and the second antibodies were added in 1/1000 with PBS for 2 h at room temperature. Finally, the HRP was added and the plates were read at 450 nm by an ELISA plate reader. The second antibodies were Rat Anti-Mouse IgE (HRP) (ab99574, Abcam, Cambridge, UK), Goat Anti-Mouse IgG1 heavy chain (HRP) (ab97240, Abcam, Cambridge, UK), and Goat Anti-Mouse IgG2a heavy chain (HRP) (ab97245, Abcam, Cambridge, UK). All experiments were performed in duplicates.

### Histological Analysis

Mice lungs were fixed with 10% formaldehyde and embedded in paraffin. Lung tissues were then cut into micro-slices, and stained with hematoxylin and eosin (H&E) or Alcian blue-periodic acid–Schiff (AB-PAS) for histological analysis. Inflammatory scores and mucus scores were graded (0 = no inflammation to 4) as past studies described [15, 16].

### SDS-PAGE and Western Blot

BPE was absorbed to NuPAGE LDS Sample Buffer (Invitrogen, Carlsbad, CA, USA). The final concentration was adjusted to 1 mg/ml with PBS. Ten microliters of BPE was used for Western blotting experiments. Birch pollen protein was fractioned by sodium dodecyl sulfate-polyacrylamide gel electrophoresis (SDS-PAGE) (Invitrogen, Carlsbad, CA, USA). Then, the gel was transferred onto a polyvinylidene fluoride (PVDF) membrane. The membrane was treated with 5% skim milk for 2 h to block nonspecific binding and incubated with the mice serum pool overnight at 4 °C (mice’s serum pool was diluted tenfold with 5% skim milk). Then the membrane was incubated with 1:1000 the second antibodies rat anti-mouse IgE (HRP) (Abcam, Cambridge, UK) for 1 h at room temperature. The procedure is described in detail by Lämmli [17].

### Mass Spectrometry

SDS-PAGE gels were selected from the Coomassie-stained gels. Each was covered with 100 μL of 50 mM ammonium bicarbonate (ABC) buffer in 50% acetonitrile (ACN) with 50 mM dithiothreitol (DTT). Each gel spot was covered with 100 μL of 50 mM ammonium bicarbonate (ABC)/50% ACN containing 50 mM iodoacetamide (IAA) and sonicated for 5 min. After 15 min, the supernatant was discarded and replaced with 100 μL of 50 mM ABC/50% ACN containing 50 mM DTT. The supernatant was discarded, and the samples were again sonicated for 5 min in 100 μL of HPLC/MS-grade water. The water was discarded, and the samples were again sonicated for 5 min in 100 μL of ACN. The ACN was discarded, and the sample-containing microtubes were air-dried to remove the remaining ACN. Next, trypsin in 10 μL of 50 mM ABC was added to each sample, and the samples were incubated overnight at 37 °C followed by the addition of trifluoroacetic acid (TFA) and ACN to final concentrations of 1% and 30%, respectively. Each sample was assayed in triplicate. LC-MS/MS analysis was performed on a Q-Exactive mass spectrometer (Thermo Fisher Scientific, Waltham, MA, USA) equipped with a nano-liquid chromatography system (Thermo Scientific EASY-nLC 1000 System, Waltham, MA, USA).

### Database Searching and Protein Identification

The raw data were extracted by ProteoWizard version 3.0.8789. All MS/MS samples were analyzed using Mascot (version 2.6.0). Mascot was set up to search the SWISS-PROT 20171211 database assuming the digestion enzyme as trypsin. Mascot was searched with a fragment ion mass tolerance of 0.020 Da and a parent ion tolerance of 10.0 PPM. Carbamidomethyl of cysteine were specified in Mascot as fixed modifications. Oxidation of methionine was specified in Mascot as variable modifications. Scaffold Q+ (version Scaffold_4.6.2) was used. Protein identifications were accepted if they could be established at greater than 90.0% probability to achieve a false discovery rate (FDR) less than 10.0% and contained at least one unique peptide.

### Statistical Analyses

Experimental data are presented as mean ± SD. Line graphs were used to show measurement metrics. Statistical differences were assessed with Mann–Whitney *U* test. We performed our analyses using the statistical software SPSS19.0 and GraphPad prism 5.0. *P* values < 0.05 were considered statistically significant.

## RESULTS

### SDS-PAGE Analysis

**Fig. 4:**
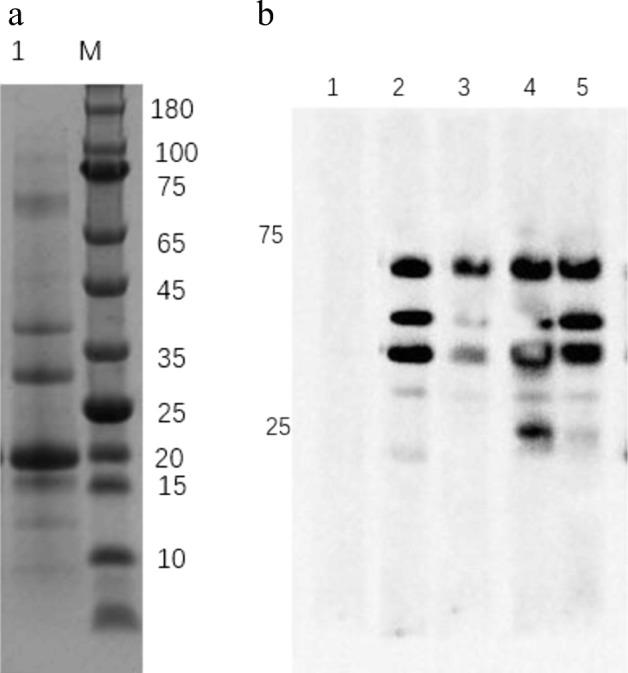
SDS-PAGE and Western blot analysis. **A** Representative image of SDS-PAGE. Lane M: molecular weight markers (180 kDa, 100 kDa, 75 kDa, 65 kDa, 45 kDa, 35 kDa, 25 kDa, 20 kDa, 15 kDa, 10 kDa). Lane 1: 10-μl protein profiles of birch pollen extracts. **B** Western blot analysis of the antigen-binding characteristics of serum specific IgE antibodies with birch pollen using sera. Lane 1: immune-blotting patterns of protein extracts with the PBS-sensitized and challenged mice serum (negative control). Lane 2: immune-blotting patterns of protein extracts with the BPE-sensitized and challenged but received PBS treatment mice serum. Lane 3: immune-blotting patterns of protein extracts with the short-term treatment group mice serum. Lane 4: immune-blotting patterns of protein extracts with the long-term treatment group mice serum. Lane 5: immune-blotting patterns of protein extracts with the observation group mice serum.

For further characterization of the birch pollen protein, we used SDS-PAGE to explore Chinese birch pollen extract. Birch pollen extracts (BPE) showed multiple protein bands with an apparent molecular mass ranging from 10 to 100 kDa and protein bands showed especially clear at 17 kDa, 28 kDa, 36 kDa and 70 kDa (Fig. 4A).

### Birch Pollen-Induced Mice Model

Compared with a PBS control group, BPE sensitized and challenged BALB/c mice (the asthma group) developed AHR, especially at methacholine concentrations of 25 mg/mL and 50 mg/mL (*P* < 0.01) (Fig. 1B). In BALF, Th2-related cytokines IL-4, IL-5, and IL-6; Th17-related cytokines IL-17; and Treg-related IL-10 and TGF-β were significantly higher in the asthma group than in the control group, while Th1-type-related IFN-γ showed no difference (Fig. 1C). As described above, mice sensitized by BPE demonstrated increased plasma levels of anti-BPE IgE, IgG1, and IgG2a (Fig. 1E). Histologic analyses showed that asthma mice lung had peribronchial infiltration of inflammatory cells and mucosal hyperplasia (Fig. 1F).

### Birch Pollen with or Without Adjuvant Suppresses Airway Allergy Inflammation

Recent studies have shown that SCIT may effectively alleviate allergy symptoms [18]. To elucidate the efficacy of SCIT, we administered Chinese birch pollen extract for the long-term treatment group. Also, we questioned whether birch pollen without adjuvant (alum) is sufficient to trigger a series of immune responses. We administered another short-term immunotherapy.

Interestingly, AHR relieved significantly in the long-term immunotherapy group than PBS treatment group (Fig. 2C). There were no differences between two groups in AHR (Fig. 2C). Compared to the PBS treatment group, the concentrations of Th2-type-related cytokines IL-4, IL-5, and IL-6 and Th17 cytokines IL-17 showed a significant reduction in the BALF in both groups. However, Treg-related IL-10 and TGF-β showed no difference in BALF only in the long-term immunotherapy group. This may be due to the limitation of the treatment period. There was no difference in Th1-type-related IFN-γ between groups (Fig. 2D) BPE-specific IgE in the short-term treatment group was lower than the PBS treatment group just as the long-term treatment group, but it was not adequate to increase the concentration of protective antibody BPE-specific IgG2a. The long-term treatment group had a higher level of BPE-specific IgG2a than other groups (Fig. 2F). Pathological inflammatory scores and mucus scores decreased significantly in long-term treatment group (Fig.2G).

### SCIT Provides Long-Term Therapeutic Benefit

The above observation led us to conclude that SCIT may inhibit airway inflammation effectively, resulting in the alleviation of the Th2-type and Th17-type immune response, but there is no evidence whether the efficacy will last for a long time. After the long-term treatment, some of the mice did not die and instead they received no treatment for 5 weeks. Then, the remaining mice were exposed to BPE aerosols again, the efficacy of SCIT remained (Fig. 3B). There is no difference between the long-term group and the observation group. However, the two groups are both significantly lower than the PBS treatment group in the level of cytokines, birch pollen specific immunoglobulins, and mucus score (Fig. 3C–F).

### Serum IgE Reactivity in Mice

Using serum from all groups of mice, IgE-binding proteins ranged from 10 to 100 kDa. The PBS-sensitized and challenged mice reacted no pollen protein, (Fig. 4B, Lane 1); the BPE asthma mice reacted strongly to 36, 45, and 70 kDa while reacted weakly to 17 and 28 kDa (Fig. 4B, Lane 2). The short-term treatment group mice (Fig. 4B, Lane 3), the long-term treatment group mice (Fig. 4B, Lane 4), and the observation group mice (Fig. 4B, Lane 5) all reacted to the 70-kDa bands.

### Mass Spectrometry

**Fig. 5:**
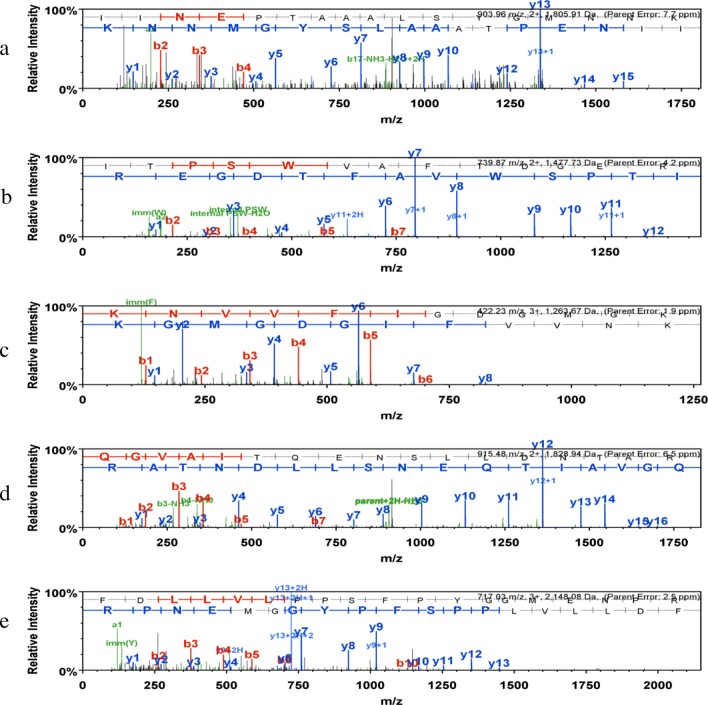
MS spectra of related proteins determined by LC-MS/MS. **A** Heat shock 70-kDa protein (HSP70_MAIZE). **B** Luminal-binding protein (BIP_SPIOL). **C** Probable pectin esterase (PME64_ARATH). **D** Glucose-6-phosphate isomerase 1 (G6PIP_ARATH). **E**UDP-sugar pyrophosphorylase (USP_ORYSJ).

To identify the 70-kDa protein in Chinese birch pollen extracts, bands were numbered, excised, in-gel digested with trypsin, and analyzed by LC-MS/MS. There are three technical repeats for each sample. Taking into account the peptide segment of the mass spectrum, we designated five proteins in database searches, as summarized in Table 1. The proteins included Luminal-binding protein, Heat shock 70-kDa proteins, probable pectin esterase, glucose-6-phosphate isomerase 1, and UDP-sugar pyrophosphorylase. The spectra of these isolated proteins from birch pollen are shown in Fig. 5A–E.

**Table 1:**
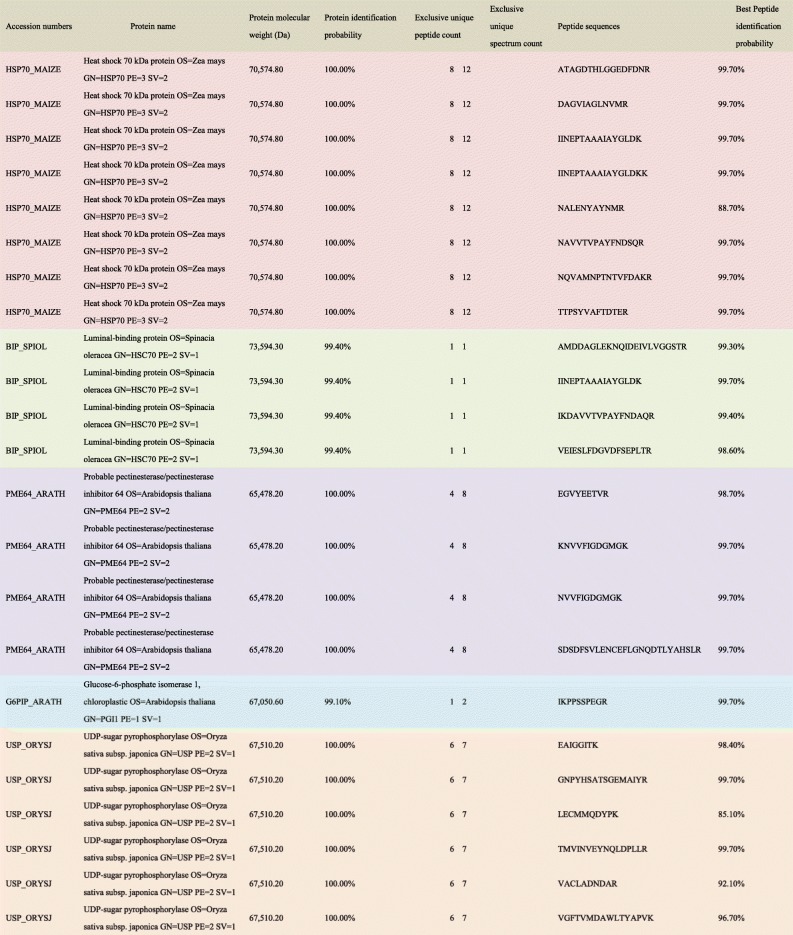
The 70-kDa Protein and Peptide Sequences in Birch Pollen Extracts Identified by Mass Spectrometry

## DISCUSSION

This is the first time that Chinese birch pollen immunotherapy was studied in mice [14, 19]. Adjuvants have been widely used to increase the immunogenicity of accompanying antigen in treating allergic diseases. Alum, the first-generation adjuvant, plays a significant role in SCIT. However, the period of BPE SCIT currently containing adjuvants (alum) is too long, leading to patients being reluctant to receive specific immunotherapy. Many adverse events of adjuvants have been reported such as chronic aluminum toxicity.

In addition to the long-term treatment group, the short-term non-adjuvant therapy group was also used in our study. The results suggest that, in the long-term treatment group, the Th2 immune response and Th17-related inflammation can be inhibited and the production of IgG blocking antibodies can be induced, thus alleviating the symptoms. Interestingly, in the short-term treatment group, there was a sufficient suppression of airway inflammation in the mice. The short-term treatment is a considerate choice for patients who are concerned about the side effects of the adjuvant (alum) in receiving BPE SCIT, but this needs further clinical studies.

Recent studies have shown that SCIT may effectively alleviate allergy symptoms [14, 20, 21], but there is no evidence as to whether the efficacy will last long term in mice. The clinical efficacy of immunotherapy is associated with symptom relief and immune homeostasis. After SCIT, the mice were re-challenged with 0.1% birch pollen aerosol to observe the immune response. We found that specific immunotherapy has a long-term therapeutic benefit.

Cytokines in BALF are measured by xMAP technology on the basis of the high flux multifactor test platform Luminex200 ™ Merck Millipore (USA) together with Luminex ® is applied in many studies to detect biomarkers [22–25]. We monitored the changes of IFN-γ, IL4, IL5, IL6, IL10, IL17, and TGF-β during the process of BPE SCIT, to find response biomarkers of birch pollen immunotherapy. Obviously, we need further studies to elucidate the immunologic mechanisms of these cytokines and their biological effects on immune cells.

We found that a 70-kDa protein played a role in a birch pollen mouse model. To gain insight into the protein content, we evaluated the protein in extracts of pollen of Chinese origin by MS-based proteomics. The heat shock protein 70 (Hsp 70) family is considered unglycosylated and exhibits an oral allergy syndrome to plant foods [26]. For example, luminal binding protein (BiP) belongs to the Hsp 70 family and plays an important role in protein synthesis and in the protection of cellular structures [27, 28]. This protein has cross-reactive homologs in many plant tissues and species and is recognized as a panallergen [1].

Our study has many limitations. On the one hand, the luminal binding protein (BiP) is just verified in birch pollen-induced mice models not in human serums, which will be improved by our laboratory in the further studies. On the other hand, we ignored to observe parts of the cytokines (IFN-gama, IL-4, IL-10, and IL-17) in BALF of long-term observation group. In that group, we focus more on the efficacy of SCIT (such as the level of AHR and sIgE) than the change of cytokines.

In conclusion, we established the first mice model of Chinese birch pollen allergy and administered Chinese birch pollen-specific immunotherapy. We compared the efficacy of SCIT with or without adjuvant alum. Moreover, a 70-kDa protein belonging to the Hsp 70 family may play a role in Chinese birch pollen allergy.
